# Linalool, citral, eugenol and thymol: control of planktonic and sessile cells of *Shigella flexneri*

**DOI:** 10.1186/s13568-018-0634-z

**Published:** 2018-06-25

**Authors:** Moisés Tomás Ngome, José Guilherme Lembi Ferreira Alves, Ana Cristina Freitas de Oliveira, Patrícia da Silva Machado, Olga Lucía Mondragón-Bernal, Roberta Hilsdorf Piccoli

**Affiliations:** 1 Laboratory of Bioprocess Engineering, Department of Food Science, Federal University of Lavras, B.O.: 3037, Lavras, MG 37.200.000, Brazil; 2 Laboratory of Food Biochemistry, Department of Food Science, Federal University of Lavras, B.O.: 3037, Lavras, MG 37.200.000, Brazil; 3 Laboratory of Food Microbiology, Department of Food Science, Federal University of Lavras, B.O.: 3037, Lavras, MG 37.200.000, Brazil

**Keywords:** Antimicrobial activity, Essential oils, Synergism, Pathogenic bacteria

## Abstract

The antimicrobial activity of linalool, citral, eugenol and thymol was determined in growth studies of both planktonic (PC) and biofilm cells (BC) *Shigella flexneri*. These components were evaluated either in isolation or in combinations using a sequential experimental strategy with Plackett & Burman and central composite rotational designs totaling 47 treatments. The minimum inhibitory concentration for PC was 0.125% (v v^−1^) for linalool and 0.5% (v v^−1^) for citral, eugenol and thymol. The biofilm minimum bactericidal concentration was 3 and 1% (v v^−1^) for linalool and citral, respectively, and 2% (v v^−1^) for eugenol and thymol. In the mixtures, the minimum concentrations in the efficient assays for PC growth inhibition were 0.0003, 0.0443 and 0.0443% (v v^−1^), for linalool, citral and thymol, respectively. In the BC, only two assays with concentrations of 0.0558, 0.0558 and 0.319% (v v^−1^) and 0.035, 0.035 and 0.3999% (v v^−1^) for linalool, citral and thymol, respectively, inhibited *Shigella* growth. Synergism was observed among the components, where PC and BC growth inhibition occurred at lower concentrations than those noted individually. The bactericidal effect of the components in microplate was different from the observed in stain steel coupons. Therefore, the obtained model can describe and predict the PC count of *S. flexneri* in medium with the tested compounds and they could be an alternative for the use in microbiological control in food industry.

## Introduction

*Shigella* spp. are rod-shaped Gram-negative bacteria belonging to the family Enterobacteriaceae (Kane and Dorman 2012). The genus *Shigella* comprises four subgroups traditionally known as species: *Shigella boydii*, *S. sonnei, S. dysenteriae* and *S. flexneri* (Cruz et al. 2014; Nüesch-inderbinen et al. 2016; Grimont et al. 2007). Among the enteric pathogenic bacteria transmitted by food, *Shigella* is one of the most common (Hu and Wai 2017). They are pathogens responsible for severe diarrheal diseases in young children worldwide, especially in developing countries (Kane and Dorman 2012; Mani et al. 2016). *Shigella* spp. can invade the mucosa of the large intestine in humans, causing inflammation and damage to the epithelium, thus giving rise to the disease called shigellosis (Arena et al. 2015). The number of deaths caused by this disease was estimated at 40,000 in 2010, whereas in 2013, 34,400 deaths of children under the age of five worldwide were estimated due to *Shigella* infections (Mani et al. 2016). In the United States, approximately 500,000 cases of shigellosis are annually reported (CDC 2013).

Foodborne illness have always been a threat to human health. They bring the emerging concern of public health in all continents, with numerous cases associated with the presence of biofilms (Srey et al. 2013).

Biofilms are associations of microorganisms attached to a surface and involved in extracellular matrix of polymeric substances (Simões et al. 2010). Any type of microorganism can form biofilm in inhospitable environments in order to survive and play an important role in several infections. The formation of biofilms is a systematic and dynamic process divided into five stages: (i) initial attachment, (ii) irreversible attachment, (iii) initial development of the biofilm architecture, (iv) maturation and (v) dispersion. The initial attachment stage is very important once it is reversible (Srey et al. 2013). In this stage, cells are called planktonic, i.e., non-adherent cells different from biofilms that have sessile cells (Costa et al. 2017).

In the food industry biofilms are generally found inside closed surfaces, such as pipes where liquid flows on solid surfaces. On open surfaces, fouling allows microbial retention (Whitehead and Verran 2015). Moreover, it is well reported that biofilm has become a problem in the food industry because it makes its population resistant to antimicrobial agents and to cleaning (Srey et al. 2013) due to their particular intrinsic characteristics, thus resulting in an increasingly negative impact on the food sector (Srey et al. 2013). In literature few studies with *S. flexneri* biofilm were done.

There is a trend of reducing the use of chemical sanitizers with antimicrobial activity in the food industry due to their negative effects (Souza et al. 2014; Moradi and Sadeghi 2017). Moreover, much pressure has been imposed by consumers and legal authorities linked to the food sector mainly focused on adopting more natural alternatives in the food production chain (Beyki et al. 2014; Souza et al. 2014). In this sense, the use of essential oils and their components, which are natural products, arises as an alternative for the control of many foodborne pathogens and degrading microorganisms (Bassolé and Juliani 2012). The essential oils and their components, such as linalool, citral, eugenol and thymol (Goldbeck et al. 2014; Ait-Ouazzou et al. 2011; Chauhan and Kang 2014), e.g., have already shown to play an important role in the discovery of new antibacterial agents (Araújo et al. 2017; Hyldgaard et al. 2012; Mahdavi et al. 2017). Although the mechanism of the antimicrobial activity of oils has not been fully understood, it is known that these make the membrane of bacterial cells more permeable, causing leakage of cytoplasmic components and then cell inactivation (Chai et al. 2016).

Therefore, the aim of the present study was to evaluate the growth inhibition of planktonic cell and biofilm of *Shigella flexneri* INCQS 00152 through both isolated and combined use of linalool, citral, eugenol and thymol.

## Materials and methods

The major essential oils (EO) p.a. grade components; linalool (97% v v^−1^), citral (95% v v^−1^), eugenol (99% v v^−1^) and thymol (99% v v^−1^), were purchased from the Sigma-Aldrich company.

### Microorganism, storage and inoculum standardization

The strain used was *S. flexneri* INCQS 00152 (ATCC 12022) supplied by the Collection of Reference Micro-organisms in Sanitary Surveillance of the Oswaldo Cruz Foundation (FIOCRUZ/CMRVS—WDCM 575). Stock cultures were stored in freezing medium (15 mL glycerol, 0.5 g bacterial peptone, 0.3 g yeast extract, 0.5 g sodium chloride, 100 mL distilled water, and pH adjusted to 7.0). Cultures were reactivated by inoculating 100 μL aliquots into tubes containing 10 mL brain–heart infusion (BHI) broth and incubated at 37 °C for 24 h. The inoculum standardization performed by using a growth curve with absorbance monitoring (OD_600nm_) of the tryptone soy broth (TSB) culture in a spectrophotometer (BEL SP-200) and plating in tryptic soy agar (TSA). The plates were incubated at 37 °C for 24 h and cultures were standardized at 10^8^ CFU mL^−1^.

### Planktonic cell death curve of *S. flexneri* exposed to major components

Exposure time influence of *S. flexneri* at different concentrations of linalool, citral, eugenol and thymol was determined through the absorbance (OD_600 nm_) monitoring in a spectrophotometer (uv BEL SP-200) every 1.0 h. Aliquots of 1 mL of standard cultures were inoculated into a flask containing 100 mL TSB plus 0.5% Tween 80 and different concentrations of major components [0.0, 0.0625, 0.125 and 0.5% (v v^−1^)]. Cultures were incubated at 37 °C for 8 h. The experiment was performed in triplicate with three repetitions.

### Selection of the main major components against planktonic cells of *S. flexneri*

The selection of the major components with greater antimicrobial effectiveness against planktonic cells (PC) of *S. flexneri* was performed using the Plackett & Burman design (Rodrigues and Lemma 2012), with eight treatments (PB 8) and 3 central points, according to Table 1.

**Table 1:** Plackett & Burman 8 matrix containing coded and real values

Test	Coded values				Real values (v v^−1^)			
	X_1_	X_2_	X_3_	X_4_	Linalool	Citral	Eugenol	Thymol
1	+ 1	− 1	− 1	+ 1	0.0156	0	0	0.0625
2	+ 1	+ 1	− 1	− 1	0.0156	0.0625	0	0
3	+ 1	+ 1	+ 1	− 1	0.0156	0.0625	0.0313	0
4	− 1	+ 1	+ 1	+ 1	0	0.0625	0.0313	0.0625
5	+ 1	− 1	+ 1	+ 1	0.0156	0	0.0313	0.0625
6	− 1	+ 1	− 1	+ 1	0	0.0625	0	0.0625
7	− 1	− 1	+ 1	− 1	0	0	0.0313	0
8	− 1	− 1	− 1	− 1	0	0	0	0
9	0	0	0	0	0.0078	0.0313	0.01563	0.0313
10	0	0	0	0	0.0078	0.0313	0.01563	0.0313
11	0	0	0	0	0.0078	0.0313	0.01563	0.0313

x_1_, x_2_, x_3_ e x_4_—codified values of linalool, citral, eugenol and thymol respectively

The solutions were prepared in TSB added with 0.5% Tween 80 and different concentrations of linalool, citral, eugenol and thymol (Table 1). Aliquots of 10 μL of standardized culture were transferred into 150 μL of solutions contained in the polystyrene microplates and incubated at 37 °C for 24 h. Subsequently, aliquots of each culture were plated in TSA then incubated at 37 °C for 24 h.

### Determination of the minimum bactericidal concentration (MBC) of mixtures of the major components on planktonic cells

The MBC of the mixtures of linalool, thymol and citral were determined using the central composite rotational design (CCRD) of 2^3^ with four center points and six axial points, totaling 18 assays. The independent variables were concentration of linalool, citral and thymol. The relationship between the coded and real values of the independent variables are presented in Table 2. The response variable was CFU mL^−1^.

**Table 2:** CCRD matrix with coded and real values of the major components for evaluation of the *S. flexneri* growth as planktonic cells (PC)

Variables	Code	Concentration (% v v^−1^)				
		− 1.68	− 1	0	1	+ 1.68
Linalool	X_1_	0.0003	0.0027	0.0063	0.0099	0.0124
Citral	X_2_	0.0001	0.0113	0.0278	0.0443	0.0555
Thymol	X_3_	0.0001	0.0113	0.0278	0.0443	0.0555

The experiment was carried out by using 96-well polystyrene microplates. The solutions were prepared in TSB added with 0.5% Tween 80 and different mixtures of major constituent mixtures (Table 2). Aliquots of 10 μL of standard culture were inoculated into the microwells containing the solutions and incubated at 37 °C for 24 h. After incubation, aliquots of the cultures were plated in TSA and incubated at 37 °C for 24 h.

### Formation of *S. flexneri* biofilm in microplates

The methodology of Vukovic et al. (2000) was used to evaluate biofilm formation capacity and the optical density of the biofilm (OD) and the optical density of the negative growth control (ODc) in microplates were measured. Aliquots (50 μL) of the cultures were inoculated into 96-well polystyrene microplates containing 150 μL the tryptone soy broth (TSB) and incubated at 37 °C for 48 h. The final values of optical density were obtained by the arithmetic means of the absorbances read at OD 600 nm in a spectrophotometer (TECAN Infinity^®^ M200 PRO) operated by the I-control^®^ software version 3.37. The essays were performed in triplicate with eight repetitions. In order to determine the biofilm formation capacity the following classification was used: OD ≤ ODc = no biofilm producer; ODc < OD ≤ 2 × ODc = weak biofilm producer; 2 × ODc < OD ≤ 4 × ODc = moderate biofilm producer; OD > 4 × ODc = strong biofilm producer.

### Biofilm minimum bactericidal concentration (BMBC) of major components

Linalool, citral, eugenol and thymol were added to the microplates containing *S. flexneri* biofilms at different concentrations. Solutions were prepared in sterile distilled water containing 0.5% Tween 80 and put in vortex for 2 min. Aliquots containing 200 μL solutions at concentrations of 0.50, 1.00, 1.50, 2.00, 2.50, 3.00, and 3.50% (v v^−1^) were added to the culture wells. After 20 min of contact, the solutions were removed and the wells were washed three times with saline solution (0.85% w/v). Then, 200 μL of TSB were added to the wells and the microplates were incubated at 37 °C for 24 h. After incubation, 10 μL of culture was plating in TSA with incubation at 37 °C for 24 h. The BMBC of major components were considered those in which there was no TSA growth after incubation. The experiment was performed in triplicate.

### Assessment of the minimum bactericidal concentration of major component combinations on *S. flexneri* biofilm (BMBC)

The CCRD of 2^3^ was carried out with six axial points and four center points, totaling 18 experiments, being evaluated three independent variables: concentration of linalool, citral and thymol (Table 3). The response variable was log CFU mL^−1^.

**Table 3:** CCRD matrix with coded and real values of the major components for determination of the minimum bactericidal concentration for *S. flexneri* biofilm growth

Variables	Code	Concentration (% v v^−1^)				
		− 1.68	− 1	0	1	+ 1.68
Linalool	X_1_	0.0001	0.0142	0.0350	0.0558	0.0699
Citral	X_2_	0.0001	0.0142	0.0350	0.0558	0.0699
Thymol	X_3_	0.0001	0.0810	0.2000	0.3190	0.3999

The solutions of the major components were prepared in sterile distilled water of 0.5% Tween 80 and put in vortex for 2 min. Aliquots of 200 μL of the component mixture solutions were added into the wells. After 20 min of contact, the solutions were removed, the wells were washed three times with saline solution (0.85% w/v) and added with 200 μL of TSB. Microtiter plates were incubated at 37 °C for 24 h, then plated in TSA and incubated again at 37 °C for 24 h. Combinations of major components where there was not growth in plates were considered as BMBC.

### Evaluation of the antimicrobial activity of the major components on *S. flexneri* biofilm formed on stainless steel coupons

*Shigella flexneri* biofilms were formed on coupons of stainless steel AISI 304 (1 × 8 × 18 mm) #4, previously sanitized and sterilized. The coupons were immersed into Petri dishes (20 × 100 mm) containing 40 mL TSB in and inoculated with 1 mL of the standardized culture of *S. flexneri*. Incubation with stirring of 25 rpm was performed at 37 °C for 48 h. After incubation, the coupons were removed, washed three times with saline solution to remove the unattached cells and immersed into solutions of major constituent mixtures for 20 min. Solutions were prepared in sterile distilled water added with 0.5% Tween 80 and major components at the concentrations generated according to the CCRD matrix of Table 3. The second washing, was then performed to remove the major components followed by the addition of TSB and incubation for 24 h. After incubation, the biofilm-borne cells were taken through swab smear. Next, the swabs were transferred to saline solution, stirred and then aliquots of the bacterial suspensions were plated on TSA and incubated at 37 °C for 24 h. The result was presented in CFU by 144 mm^2^.

The antimicrobial action of the different solutions containing the major components linalool, citral and thymol was compared with 3% v/v quaternary ammonium (Sandet) action, a widely used commercial chemical disinfectant in the food industry.

### Statistical analysis

The Statistica 8.0 software (Statsoft Inc, 2008**)** was used for the statistical analysis of all experimental results in this paper, with 5% significance for CCRD and for the essays of the reduction of *S. flexneri* populations in the coupons and with 10% significance for PB design.

## Results

**Fig. 1:**
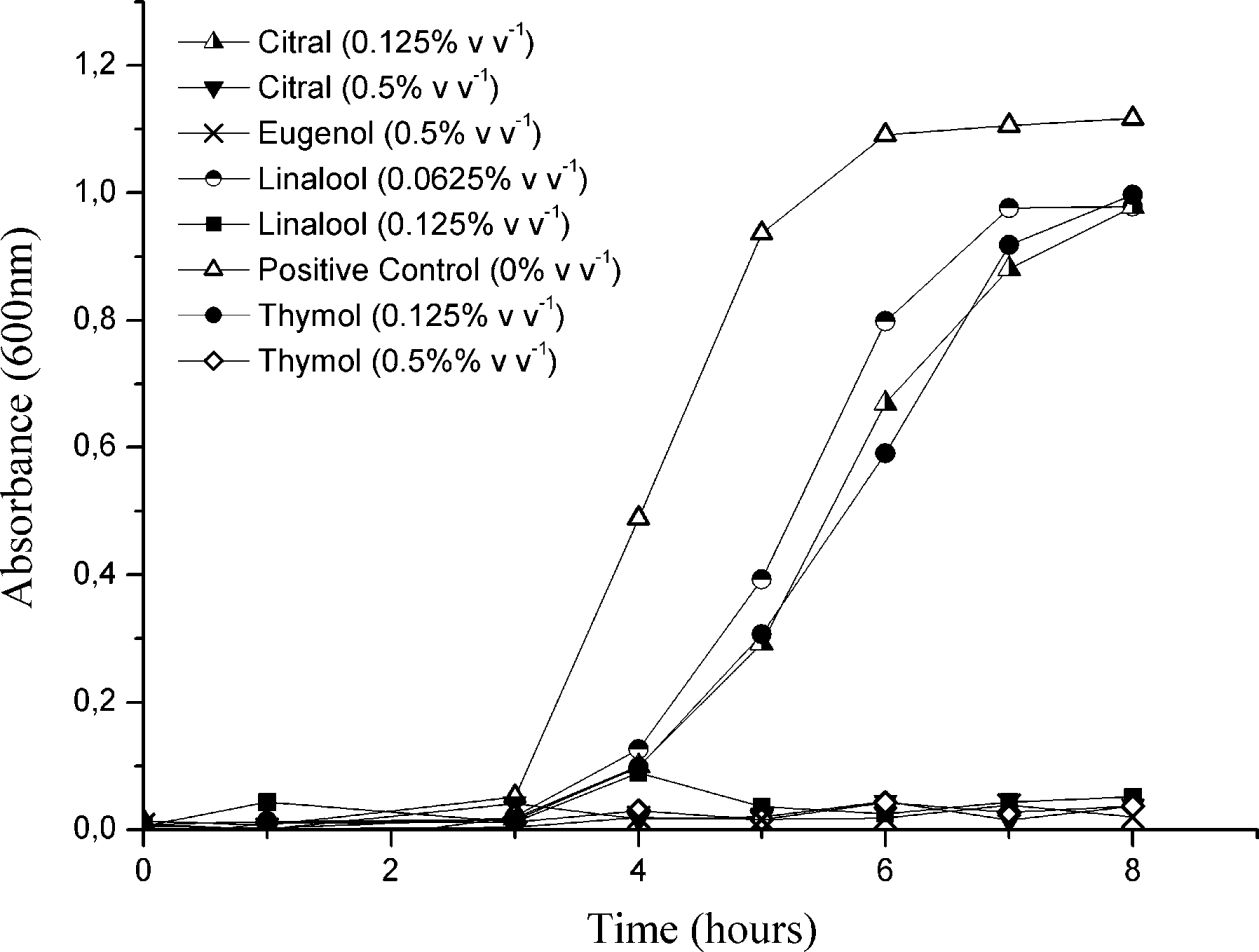
Planktonic cell growth curve of *S. flexneri* in medium with EO major components

The antimicrobial activity of essential oil major components was evaluated against *S. flexneri* through absorbance as a function of time in hours. Figure 1 shows the growth/death curves of *S. flexneri* in medium at different concentrations of linalool, citral, eugenol and thymol, incubated for 8 h at 37 °C.

All analyzed compounds showed bactericidal activity on *S. flexneri*. Linalool was the component with the highest bactericidal activity, inhibiting the growth of *S. flexneri* at the concentration of 0.125% (v v^−1^). For the other components, the bactericidal activity was observed at the concentration of 0.5% (v v^−1^). All components did not inhibit *S. flexneri* at concentrations below 0.0625% (v v^−1^).

**Fig. 2:**
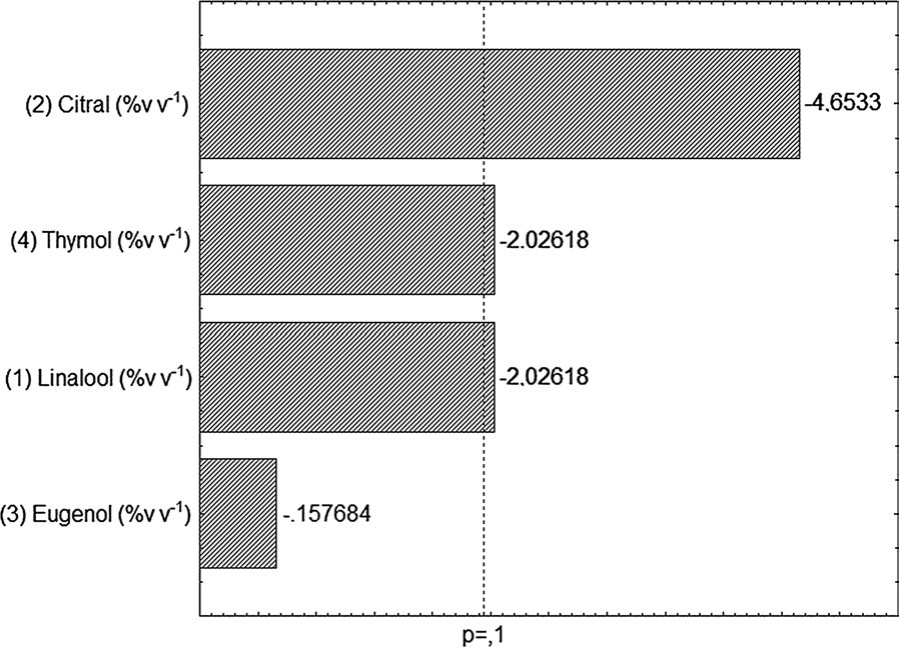
Pareto chart with estimated effect (absolute value) of the studied variables in the PB 8 experimental design for growth control of *S. flexneri*

The antimicrobial synergism among the major components on PC was initially studied using the Plackett & Burman design 8 (PB 8). There was only cell growth of *Shigella* in treatments 1, 5, 7 and 8. Pareto chart of the studied variables in the PB design for the growth of *S. flexneri* is shown in Fig. 2.

In the selection of variables, the components citral, linalool and thymol had significant negative effects, with 90% confidence level, showing that they were efficient in inhibiting PCs of *S. flexneri.* It is observed that when the concentrations of these variables increase from level − 1 to + 1, the antimicrobial activity of these components is increased. Thus, citral, linalool and thymol were selected for further experiments.

Central composite rotational designs was used to determine the best concentrations of the EOs major components for growth inhibition of *S. flexneri*. Table 5 shows the experimental data regarding *S. flexneri* cell growth in the planktonic form at different concentrations of linalool, citral and thymol.

It is observed in Table 4 that in assays 4 (0.0099 linalool, 0.0443 citral, 0.0113% v v^−1^ thymol), 7 (0.0027 linalool, 0.0443 citral, 0.0443% v v^−1^ thymol), 8 (0.0099 linalool, 0.0443 citral, 0.0443% v v^−1^ thymol), 12 (0.0063 linalool, 0.0555 citral, 0.0278% v v^−1^ thymol) and 14 (0.0063 linalool, 0.0278 citral, 0.0555% v v^−1^ thymol), there was no growth of planktonic cells incubated for 24 h at 37 °C. For the other assays, there was growth. When comparing the antimicrobial activity of assays 4, 7, 8, 12 and 14 with those of the same components, nevertheless, pure, on the PC, it is noted that the antimicrobial action occurs at concentrations about 10–50 times more diluted, since the MIC for linalool, citral and thymol were 0.125; 0.5 and 0.5% (v v^−1^), respectively.

**Table 4:** CCRD results for planktonic cells (PC) count of *Shigella flexneri* (Log CFU mL^−1^)

Test	Codified values			Real values (% v v^−1^)			Log CFU mL^−1^ Planktonics cells
	X1	X2	X3	Linalool	Citral	Thymol	
1	− 1	− 1	− 1	0.0027	0.0113	0.0113	7.3711
2	1	− 1	− 1	0.0099	0.0113	0.0113	7.8451
3	− 1	1	− 1	0.0027	0.0443	0.0113	5.8325
4	1	1	− 1	0.0099	0.0443	0.0113	0.0
5	− 1	− 1	1	0.0027	0.0113	0.0443	5.6628
6	1	− 1	1	0.0099	0.0113	0.0443	6.3345
7	− 1	1	1	0.0027	0.0443	0.0443	0.0
8	1	1	1	0.0099	0.0443	0.0443	0.0
9	− 1.68	0	0	0.0003	0.0278	0.0278	6.5038
10	1.68	0	0	0.0124	0.0278	0.0278	6.6776
11	0	− 1.68	0	0.0063	0.0001	0.0278	8.2945
12	0	1.68	0	0.0063	0.0555	0.0278	0.0
13	0	0	− 1.68	0.0063	0.0278	0.0001	8.2945
14	0	0	1.68	0.0063	0.0278	0.0555	0.0
15	0	0	0	0.0063	0.0278	0.0278	7.5185
16	0	0	0	0.0063	0.0278	0.0278	7.7243
17	0	0	0	0.0063	0.0278	0.0278	7.7634
18	0	0	0	0.0063	0.0278	0.0278	7.7482

X1, X2, X3—coded and real values of linalool, citral and thymol concentrations respectively

Multiple regression analyses were carried out for the variable CFU mL^−1^ of planktonic cells based on the experimental data presented on Table 4. Table 5 presents the regression coefficients for growth of *S. flexneri*, where the significant values (p < 0.05) are highlighted. It can be seen that the linear and quadratic terms of citral and thymol concentrations presented significant negative effects on the PC counting at 95% confidence level.

**Table 5:** Regression coefficients of planktonic cells (PC) count of *S. flexneri* (Log CFU mL^−1^)

Source of variation	Average/interaction	X_1_ (L)	X_1_ (Q)	X_2_ (L)	X_2_ (Q)	X_3_ (L)	X_3_ (Q)	X_1_ X_2_	X_1_ X_3_	X_2_ X_3_
Planktonic Cells Count (PC) (Log CFU mL^−1^)										
Regression	*7.722*	− 0.322	− 0.533	*− 2.588*	− *1.398*	− *1.685*	− *1.398*	− 0.872	0.754	− 0.327
*p* value	0.000	0.374	0.173	0.000	0.004	0.001	0.004	0.087	0.130	0.486
R^2^	*0.935*									

Significant values (*p* < 0.05) are highlighted in italics

*X*_*1*_ linalool, *X*_*2*_ citral, *X*_*3*_ thymol, *L* linear, *Q* quadratic

This means that an increase in the citral and thymol concentration reduced PC counts. Disregarding the non-significant terms, the reparameterized models were obtained for planktonic cell counts of *S. flexneri* INCQS 00152, Eq. 1.

1 $${\text{Log CFU mL}}^{ - 1} = { 7}. 1 4 9 8 { }{-}{ 2}. 5 8 8 2 {\text{x}}_{ 2} {-}{ 1}. 6 8 4 6 {\text{x}}_{ 3} {-}{ 1}. 2 8 7 2 {\text{x}}_{ 2}^{ 2} - 1. 2 8 7 2 {\text{x}}_{ 3}^{ 2}$$

**Fig. 3:**
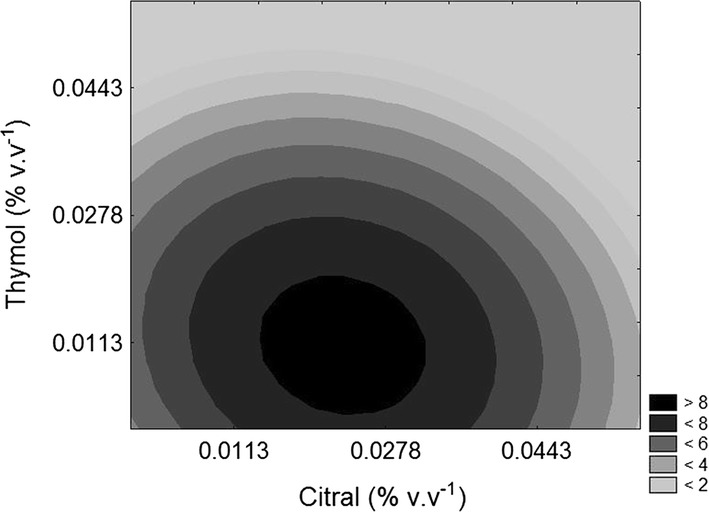
Contour curve for planktonic cells (PC) count (Log CFU mL^−1^) as a function of citral and thymol concentration (linalool concentration was fixed at the lowest level—0.0003% v v^−1^)

The ANOVA of the quadratic regression reparameterized model for the PC counts was significant (p < 0.05), since the F_calc_ regression value (18.59) was higher than the value of Ftab (F_4;13;0.05_ = 3.18). The determination coefficient (R^2^) was high (0.851). This indicated that the reparameterized model for PC counts had a good fit and could be applied for response prediction. Contour curve that describe the influence of thymol and citral on the counting of planktonic cells is shown in Fig. 3.

It is observed that when higher concentrations of citral and thymol are added to the medium (above 0.0443% v v^−1^), the lowest PC counts of *S. flexneri* are obtained. The antibacterial action of citral was higher, as can be observed by the higher coefficient of the linear term for citral in the Eq. 1 (− 2.5882) when compared to the coefficient of the linear term for thymol (− 1.6846).

Thus, for the highest possible antibacterial action on PCs of *S. flexneri*, it is suggested the addition of citral and thymol at concentrations above 0.0443% v v^−1^.

The results of the optical density of the negative growth control (ODc) and optical density of the biofilm (OD) were 0.05 and 1.05 respectively. These results of the formation of *S. flexneri* biofilm in microplates showed that this microorganism is a strong biofilm producer. Thus, experiments with *S. flexneri* biofilms were performed.

When in biofilm, *S. flexneri* was more resistant to antimicrobials, being that BMBC was 2–24 times higher in the analyzed components than those determined for PC of *S. flexneri.* The most effective component against the bacterium was citral, which BMBC was 1%. Eugenol and thymol showed the same BMBC (2%). Although linalool was the most effective one against PC, it did not show the same effect on the biofilm, being BMBC of 3% v v^−1^. Table 6 shows biofilm of *S. flexneri* cellular growth experimental data treated with different linalool, citral and thymol concentrations.

**Table 6:** Results for biofilm cells count of *Shigella flexneri* (Log CFU mL^−1^)

Test	Codified values			Real values (% v v^-1^)			Log CFU mL^−1^ Biofilm
	X1	X2	X3	Linalool	Citral	Thymol	
1	− 1	− 1	− 1	0.0142	0.0142	0.081	6.382
2	1	− 1	− 1	0.0558	0.0142	0.081	6.3139
3	− 1	1	− 1	0.0142	0.0558	0.081	6.3692
4	1	1	− 1	0.0558	0.0558	0.081	6.0645
5	− 1	− 1	1	0.0142	0.0142	0.319	6.0934
6	1	− 1	1	0.0558	0.0142	0.319	6.0492
7	− 1	1	1	0.0142	0.0558	0.319	6.0934
8	1	1	1	0.0558	0.0558	0.319	0
9	− 1.68	0	0	0.0001	0.035	0.2	7.0969
10	1.68	0	0	0.0699	0.035	0.2	5.8513
11	0	− 1.68	0	0.035	0.0001	0.2	6.3874
12	0	1.68	0	0.035	0.0699	0.2	6.2765
13	0	0	− 1.68	0.035	0.035	0.0001	7.9731
14	0	0	1.68	0.035	0.035	0.3999	0
15	0	0	0	0.035	0.035	0.2	7.7782
16	0	0	0	0.035	0.035	0.2	7.6021
17	0	0	0	0.035	0.035	0.2	7.6532
18	0	0	0	0.035	0.035	0.2	7.7634

X1, X2, X3—coded and real values of linalool, citral and thymol concentrations, respectively

In regard to biofilms, it can be observed in Table 6 that, only assays 8 (0.0558 linalool, 0.0558 citral, 0.319% v v^−1^ thymol) and 14 (0.035 linalool, 0.035 citral and 0.3999% v v^−1^ thymol) did not show growth after 20 min of exposure. These results indicate that the presence of components as linalool, citral and thymol affect the growth of biofilm cells. When comparing the antimicrobial activity of assays 8 and 14 with the components used in isolation in the BC, the antimicrobial action was observed with concentrations from 6 to 86 times more diluted, since the BMBC for linalool, citral and thymol were 3, 1 and 2% (v v^−1^), respectively. Therefore, it was observed that, the use of valid combinations allows rationalizing these natural compounds and obtaining positive results on the growth inhibition of *S. flexneri.*

The multiple regression analysis for the variable CFU mL mL^−1^ for the biofilm cells shows that the thymol concentration linear and quadratic terms of the model below the BC count showed significant negative effects at 95% confidence level.

2 $${\text{Log CFU mL}}^{ - 1} {\text{Biofilm }} = { 6}. 8 6 7 9 { } - { 1}. 4 8 6 9 {\text{x}}_{ 3} - { 1}. 1 6 3 4 {\text{x}}_{ 3}^{ 2}$$

This means that the increased concentration of thymol reduces the cell count in biofilm, within the established limits in the present study. The parameters with (p < 0.05) were considered as significant. Table 7 presents the regression coefficients for growth curve of *S. flexneri*, being highlighted the significant values (p < 0.05).

**Table 7:** Regression coefficients of biofilm cells (BC) count of *S. flexneri* (Log CFU mL^−1^)

Source of variation	Average/interaction	X_1_ (L)	X_1_ (Q)	X_2_ (L)	X_2_ (Q)	X_3_ (L)	X_3_ (Q)	X_1_ X_2_	X_1_ X_3_	X_2_ X_3_
Biofilm cells count (BC) (Log CFU mL^−1^)										
Regression	*7.702*	− 0.631	− 0.444	− 0.476	− 0.494	− *1.487*	− *1.325*	− 0.786	− 0.721	− 0.723
p-value	*0.000*	0.120	0.273	0.226	0.227	*0.003*	*0.008*	0.136	0.167	0.165
R^2^	*0.839*									

Significant values (*p* < 0.05) are highlighted in italics

*X*_*1*_ linalool, *X*_*2*_ citral, *X*_*3*_ thymol, *L* linear, *Q* quadratic

By disregarding the non-significant terms, the reparameterized model was obtained for cell counts in biofilm of *S. flexneri*. Although the ANOVA of the quadratic regression model for the BC count was significant (p < 0.05), the coefficient of determination (R^2^) was low (0.544), indicating a low model fitting to the experimental data.

In Table 6 it can be seen that in trials 8 (0.0558% v v^−1^ linalool, 0.0558% v v^−1^ citral, 0.319% v v^−1^ thymol) and 14 (0.035% v v^−1^ linalool, 0.035% v v^−1^ citral and 0.3999% v v^−1^ thymol) no growth of *Shigella* was observed in microplate. Experiments were then carried out with the same concentrations, but in stainless steel coupons. Stain steel coupons treated with *Shigella* were tested with EOs. The results showed growth of *Shigella* (5.36 ± 0.03 and 5.31 ± 0.08 in Log CFU mL^−1^), after 20 min of exposure of the major essential oils compounds. When the concentrations of assays 8 and 14 were doubled, biofilm cells growth in the coupons was not noted, the same result observed in the control containing quaternary ammonium.

Statistical analysis of the growth data of biofilm cells of *S. flexneri* in the coupons showed a significant difference between trials 8 and 14 and the same trials with duplicate concentrations (0.1116% v v^−1^ linalool, 0.1116% v v^−1^ citral, 0.638% v v^−1^ thymol) and (0.070% v v^−1^ linalool, 0.070% v v^−1^ citral and 0.7998% v v^−1^ thymol) verified by Tukey test at 5% probability.

## Discussion

There are several studies in the literature on the bactericidal effect of EO and its major components. Bagamboula et al. (2004). It was found that carvacrol showed the highest antibacterial activity against *S. flexneri, S. sonnei, E. coli* followed by thymol, whereas both estragole and linalool showed limited antibacterial activity. These authors achieved an inhibitory effect below the detection limit against Enterobacteriaceae at concentration of 0.5% (v v^−1^) of carvacrol and thymol. Thus, Korenblum et al. (2013) proved that citral was responsible for the antimicrobial effect as no inhibition difference was observed between the essential oil and its main component. Gaio et al. (2015) evaluated the antibacterial activity of basil essential oil in vitro and in Italian-type sausage and found minimal inhibitory concentration for the growth of *Shigella flesneri* of 0.75 mg mL^−1^. For all tested Gram-positive and -negative bacteria, basil essential oil presented antibacterial activity, with the exception of *Pseudomonas aeruginosa*, and the minimum inhibitory concentration varied from 0.25 to 1.00 mg g^−1^.

In the present article, linalool, citral, eugenol and thymol analyzed in isolation, inhibited the growth of *S. flexneri*. Nevertheless, as shown in Fig. 1, linalool presented a higher antimicrobial activity against the PC at a concentration of 0.125% (v v^−1^). In the same evaluation, the components citral, eugenol and thymol had the same effect in the same bacteria at 0.5% (v v^−1^). Thus, this paper suggests such EO natural compounds may comprise a new generation of intelligent antimicrobial sanitizers that can reduce the incidence of foodborne illness caused by *S. flexneri*. Therefore, the use of these compounds in the food industry as antimicrobial agents are considered promising preservatives and chemical sanitizers substitutes. Khan et al. (2017) commented that organic compounds extracted from plants are an attractive alternative to replace conventional antimicrobial agents.

There are several mechanisms involved in the antimicrobial activity of the EO major components that can begin by the cell membrane degradation, the permeability increase of the membrane, up to the decrease of cytoplasmic pH. According to Mackey and Paga (2009), the mechanism of microbial inactivation by citral seems to be a complex phenomenon involving the occurrence of different types of lesions. Exposure with citral damages the plasma and outer cell membranes. These authors verified that repair of lesioned *E. coli* cells after exposure to citral required lipid synthesis and energy expenditure. Thus, cell membrane is confirmed as being one of the structures involved in microbial inactivation by citral. It was observed Chauhan and Kang (2014) that thymol acts on the membrane integrity, promoting release of intracellular potassium ions and nucleic acids, thus causing irreversible damage of bacterial membranes. Moon and Rhee (2016) noticed that the addition of 0.5 mM thymol to soy sauce reduced the populations to < 2.0 log CFU mL^−1^ after 5 min, and to below the detection level in 10 min at 22 °C (initial populations of *Escherichia coli* O157:H7, *Salmonella Typhimurium* and *Listeria monocytogenes*a were between 7.1 and 7.3 log CFU mL^−1^).

The ability of bacteria causing food poisoning to form biofilm is already well established (Valeriano et al. 2012; Oliveira et al. 2010; Millezi et al. 2013; Zhao et al. 2017). *Shigella flexneri* strain used in the present study was strong biofilm-forming, being highlighted its importance for the food industry. In a study performed in an ice cream processing facility, both *Listeria monocytogenes* and *S. flexneri* were highlighted, forming biofilms in the processing line (Gunduz and Tuncel 2006). Sharma and Anand (2002) showed that bacteria of the genus *Shigella* were among gram-negative biofilm-forming bacteria in milk pasteurization line. Thus, the discovery of new biofilm control strategies in the food industry based on natural substances with high antimicrobial activity seems to be a step forward in overcoming the issue of biofilm resistance (Simões et al. 2010). The antibacterial activity of EO and its components is not attributed to a single element (Kim and Kang 2017). It was observed that the combinations containing linalool, citral and thymol (tests: 4, 7, 8, 12 and 14 in Table 4) showed higher antimicrobial activity against PC in relation to antimicrobial activity in isolation. The same was observed for BC in the assays 8 and 14 (Table 6). These results show the synergistic effect among the tested EO major components, playing an important role in the death of PC and BC. However, an additional study is necessary to evaluate the antibacterial action of the compounds used in this work in the case of multi-species biofilms.

Henri et al. (2012) reported synergistic effects between eugenol and thymol against *E. coli,* where a combined effect resulted in a greater reduction of the bacterial population than when applied in isolation. The authors suggest that thymol disintegrated the outer membrane, facilitating the entry of eugenol into the cytoplasm, thus denaturing proteins. Khan et al. (2017) noticed that thymol and carvacrol induce autolysis, stress, growth inhibition and reduce the biofilm formation by *Streptococcus mutans*. Other authors have found that thymol and carvacrol exhibited increased antimicrobial activity against pathogenic bacteria (*Clostridium perfringens, Escherichia coli* and *Salmonella*) than against beneficial bacteria.

Tables 5 and 7 present the linear increase in PC death and BC elimination in as much as concentrations were increased. It is clear considering Tables 4 and 6 that the concentrations of linalool, citral and thymol contributed to the removal of PC and BC. However, only the concentration of citral and thymol were significant for the removal of PC, as shown in Fig. 3 and Table 5. For BC, the thymol concentration was the variable that most contributed to their removal (Table 7). A previous study by Korenblum et al. (2013) showed that planktonic and sessile growth of *Desul fovibrio alaskensis* isolated from a soured oil reservoir was inhibited by lemongrass essential oil and its major component citral.

Assays 8 (0.0558 linalool, 0.0558 citral, 0.319% v v^−1^ thymol) and 14 (0.035 linalool, 0.035 citral and 0.3999% v v^−1^ thymol) were effective in BC control in the microtiter plate (Table 6), however, they did not show the same efficiency in BC control in the coupons. Nevertheless, double concentrations of the assays 8 and 14 for BC exposure of *S. flexneri* formed in the coupons led to reductions at undetectable levels in the bacterial populations of the biofilm. No differences were also observed among the control results (quaternary ammonium, prepared according to the manufacturer’s recommendations for BC control) and double concentrations of the assays 8 and 14 for BC.

Therefore, the naturally occurring phytochemicals studied in this paper, specially citral and timol isolated or combined could be used for microbiological control in the food industry substituting chemical compounds. The obtained model can describe and predict the PC count of *S. flexneri* in medium with the tested major compounds. The major components applied individually or in combination showed also a bactericidal action on the biofilm cells of *S. flexneri*. In mixtures they showed synergism, allowing the reduction of the concentration of the major components and chemical sanitizers with lower environmental impact and lower cost.

## Abbreviations

PC: planktonic cells
BC: biofilm cells
CCRD: central composite rotational designs
MIC: minimum inhibitory concentration
BMBC: biofilm minimum bactericidal concentration
BHI: brain–heart infusion
EO: essential oils
TSB: tryptone soy broth
OD: optical density
CFU: colony forming unit
PB: Plackett & Burman design
MBC: minimum bactericidal concentration
ODc: optical density of the negative growth control
